# Surgical Outcomes of VRAM vs. Gracilis Flaps in Vulvo-Perineal Reconstruction Following Oncologic Resection: A Proportional Meta-Analysis

**DOI:** 10.3390/cancers14174300

**Published:** 2022-09-01

**Authors:** Ebai A. Eseme, Matteo Scampa, Juan A. Viscardi, Myriam Ebai, Daniel F. Kalbermatten, Carlo M. Oranges

**Affiliations:** Department of Plastic, Reconstructive, and Aesthetic Surgery, Geneva University Hospitals, Geneva University, 1205 Geneva, Switzerland

**Keywords:** VRAM, gracilis, vulvo-perineal amputation, pelvic exenteration, surgical outcomes, meta-analysis, complications, reconstruction

## Abstract

**Simple Summary:**

The rate of perineal complications after abdominoperineal reconstruction for the treatment of cancers ranges from 25% to 60% in the literature. It is well-established in current literature that direct closure has a higher complication rate than closure with a flap. Several reconstructive options have been proposed to fill the dead space with well-vascularized tissue. Every surgeon would like to be comfortable in selecting which flap has superiority in terms of surgical outcome. In the absence of a meta-analysis on the subject due to the scarcity of RCT and comparative studies, we used a proportional meta-analysis to analyze the surgical outcomes after reconstruction with either VRAM flap or gracilis flap following oncologic resection of the vulvo-perineal region.

**Abstract:**

Pelvic exenteration and abdominoperineal resection are radical techniques commonly used for locally advanced or recurrent pelvic malignancy with high morbidity due to large pelvic defects. Flaps can help provide healthy, well-vascularized, non-irradiated tissues to fill pelvic dead space. We conducted a proportional meta-analysis to compare surgical outcomes of vertical rectus abdominus myocutaneous flap (VRAM) vs. gracilis flap for vulvo-perineal reconstruction following oncologic resection. A comprehensive literature search was conducted in the MEDLINE, PubMed, Embase, Google Scholar, and Cochrane Library databases. Proportional meta-analysis was performed to compare the surgical outcomes of using VRAM or gracilis flaps. Our review yielded 16 eligible studies. The pooled resolution rate of overall donor site complications for VRAM flap (pooled proportion = 0.576 [95% CI 0.387, 0.754]) was significantly higher than the pooled rate of overall donor site complications of gracilis flap (pooled proportion = 0.160 [95% CI 0.058, 0.295]). Partial and total flap necrosis were similar in both groups. There was no statistically significant difference between minor and major complications for both flaps. Both flaps can be used safely for vulvo-perineal reconstruction following oncologic resection with similar recipient site outcomes, although the VRAM flap will have more donor site complications than the gracilis flap.

## 1. Introduction

Pelvic exenteration and abdominoperineal resection (APR) are radical techniques commonly used for locally advanced or recurrent pelvic malignancy [1]. Although neoadjuvant chemotherapy and/or radiotherapy has been shown to increase radical resection rates, decrease local recurrence rates, and increase cancer-specific survival, the morbidity rates due to wound complications remain high [2,3]. In order to achieve negative margins, wide excisions are required, usually leaving a large residual defect [4]. This defect favors fluid accumulation, potentially leading to infections, dehiscence, and delayed healing [5,6]. The rate of perineal complications after APR is high and ranges from 25% to 60%, placing a high burden on the patient’s quality of life [7,8,9,10,11,12]. Closure of the surgical site is a complex task that should be addressed by a multidisciplinary team in a high-competency reference center. Healthy, well-vascularized, non-irradiated tissue is usually required to fill the pelvic dead space and minimize complication rates [10,13,14,15,16]. Flaps have been shown to resist bacterial infection in vivo and favor wound healing by providing a steady blood supply that delivers oxygen, immunological cells, and antibiotics to the defect site [10,15,16]. A wide variety of techniques exist to reconstruct perineal defects following oncologic resection, but muscular pedicled flaps have gained interest among professionals due to their robust blood supply, the absence of microsurgical anastomosis, and their ability to offer tension-free closure [1,17,18]. Among them, VRAM and gracilis are two popular options [1,19,20,21]. It is in every surgeon’s interest to offer the best approach to their patients, aiming at positively impacting their quality of life. A comparison between both flaps has not been sufficiently assessed in the current literature to define which reconstruction provides the best surgical outcomes.

Our aim is to synthetize all available evidence on VRAM and gracilis flap usage for vulvo-perineal reconstruction following oncologic resection to identify potential differences in postoperative surgical outcomes and help guide practicians in selecting an adequate reconstruction strategy. Due to the limited availability of comparative studies, a traditional meta-analysis was not deemed possible. Instead, a proportional meta-analysis approach was chosen, using the method of treatment as a moderator to evaluate the differences in baseline patients’ characteristics and selected outcome ratios [22].

## 2. Materials and Methods

This meta-analysis follows the Preferred Reporting Items for Systematic Reviews and Meta-Analysis (PRISMA) recommendations.

*Systematic review*: A comprehensive systematic review of the published literature on Embase, PubMed, and Cochrane Library was performed in April 2022, aiming at all studies focusing on surgical outcomes after VRAM or gracilis reconstruction following oncologic perineal resection. The keywords VRAM, gracilis, perineal oncologic resection, and abdominoperineal resection were used as search strings using Boolean operators. An additional secondary manual query on Research Gate and Google Scholar was conducted to retrieve articles potentially missed during the systematic review. Furthermore, selected articles’ references were assessed to identify potentially relevant articles.

*Inclusion and exclusion criteria*: The selection process aimed at all articles considering pedicled VRAM or/and gracilis flaps for vulvo-perineal reconstruction after oncologic resection. Pelvic exenteration was defined as the resection of any/all visceral structures in the anterior pelvic cavity with or without proctocolectomy and excluded all bony resections. Selected articles had to report postoperative outcomes for each intervention group. Postoperative clinical outcomes investigated included overall complication rate for donor and recipient site. Then, further precision was sought by assessing perineal infection rate, partial and total flap failure, dehiscence rates, and infection rates. Studies were deemed eligible if one or more of these pre-specified outcomes were reported. Inclusion/exclusion criteria are further detailed in Table 1. Case reports and small case series (<10 patients) were excluded, as were commentaries, letters to editors, cadaveric studies, animal studies, and articles not written in English.

*Selection process and data extraction*: Titles and abstracts were independently scrutinized using Rayyan software by two independent reviewers (E.E. and M.S.) to identify all potentially relevant articles [23]. Doubtful cases were included for complete reading to avoid missing potentially eligible articles.

Selected articles were then fully read by two independent authors (E.E. and M.S.). If they met all selection criteria, data were extracted on a standardized Excel file by both authors and compared for similarity. If divergence or doubt subsisted, consultation with a senior author (C.M.O.) allowed resolution of the issue. Data collection aimed at study characteristics, patient demographics, comorbidities, oncologic diagnosis, operative details, and surgical outcomes. There was no attempt to retrieve missing data from the authors.

*Statistical analysis*: Articles were dichotomized in two groups: VRAM or gracilis. Data were processed through the JBI SUMARI web application on 10 May 2022 to conduct the proportional meta-analysis of surgical outcomes [24]. Pooled proportions were estimated using the Freeman–Tukey transformation with a random effect model. Results were expressed in proportions with a 95% confidence interval. Heterogeneity was tested using the I2 statistic, with values lower than 30% defined as low heterogeneity, between 30 and 70% as intermediate, and more than 70% as high heterogeneity [25]. Differences in pooled proportions were then compared for statistical significance using a two-tailed Z-test. A *p*-value of less than 0.05 was considered statistically significant.

## 3. Results

**Figure 1:**
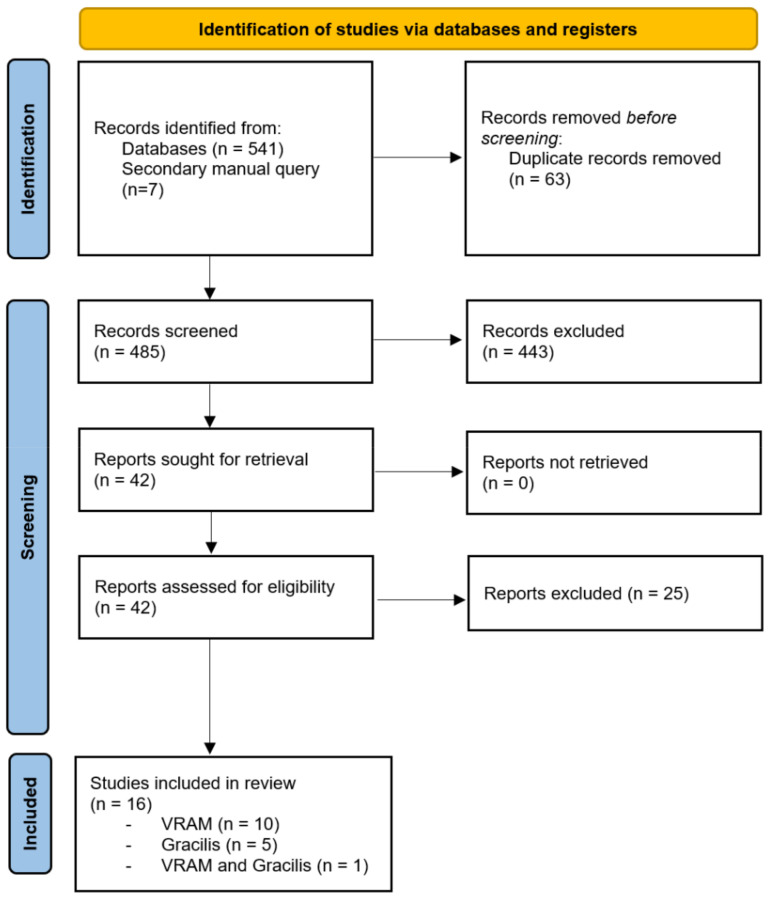
PRISMA flow chart.

The initial search query resulted in 548 citations; after removing duplicates (63 citations), 485 titles remained. Full-text review was done for 42 articles after the initial screening. Sixteen studies were identified, one of which, from Stein et al., separately reported surgical outcomes for VRAM and gracilis [26]. There were 5 prospective studies and 11 retrospective studies [11,12,21,26,27,28,29,30,31,32,33,34,35,36,37,38] (Figure 1).

### 3.1. Study and Patient Characteristics

The 16 studies were conducted in Europe (*n* = 6), the United States (*n* = 7), Australia (*n* = 1), Africa (*n* = 1), and Canada (*n* = 1) and were published between 1994 to 2020 [11,12,21,26,27,28,29,30,31,32,33,34,35,36,37,38]. One study was multicentric and 15 were monocentric. A total of 925 patients were included in our study for analysis with 783 VRAM (84.3%) and 142 gracilis (15.7%) (Table 2).

**Table 2:** Summary of study characteristics.

Reference	Study Design	Center	Total No. of Patients	Mean Age	Type of Cancer	Type of Resection	Type of Flap	Mean Duration of Follow-Up (Mo)
**Lefevre 2009** [32]	Observational Prospective	Monocenter	43	59.5 ± 12.8	Anal	APR	VRAM	40.7 (1.5–174.3)
**Bell 2004** [11]	Observational prospective	Monocenter	55	55 (30–77)	Anal	APR	VRAM	9 (1–27)
**Barker 2013** [27]	Observational retrospective	Monocenter	55	65 (38–84)	Anal, rectal	APR	VRAM	NS
**Combs 2014** [28]	Observational retrospective	Monocenter	49	54.7 ± 11.7	Anal, rectal	APR	VRAM	25.6 ± 29.3
**Van Ramshorst 2020** [29]	Observational prospective	Monocenter	87	60 (51–66)	Anal, rectal	APR	VRAM	20 (8–39)
**Harries 2021** [30]	Observational retrospective	Monocenter	173	67 (28–88)	Rectal	APR	VRAM	NS
**Touny 2014** [31]	Observational prospective	Monocenter	30	53.3 (26–68)	Anal	APR	VRAM	NS
**Buchel 2004** [12]	Observational retrospective	Monocenter	73	56.1 (28–79)	Anal	APR	VRAM	NS
**Stein 2018** [26]	Observational retrospective	Multicenter	88	62.4 (11.6)	Diverse tumors and non-oncologic etiologies	APR	VRAM	NS
**McMenamin 2011** [33]	Observational retrospective	Monocenter	16	63.6 (29–83)	Diverse tumors and non-oncologic etiologies	APR	VRAM	NS
**Nelson 2008** [34]	Observational prospective	Monocenter	114	58.0 ± 12.0	Diverse tumors and non-oncologic etiologies	NS	VRAM	24.2 20.6
**Chong 2005** [35]	Observational retrospective	Monocenter	16	62 ± 8 (53–78)	Diverse tumors	NS	Gracilis	NS
**Coelho 2019** [36]	Observational retrospective	Monocenter	25	62 (26–80)	Anal, prostate, rectal	NS	Gracilis	19 (3–102)
**Sheckter 2016** [37]	Observational retrospective	Monocenter	16	57.94	Diverse tumors and non-oncologic etiologies	APR	Gracilis	35 ± 26.8
**Stein 2018** [26]	Observational Retrospective	Multicenter	27	57 (10.7)	Diverse tumors and non-oncologic etiologies	APR	Gracilis	NS
**Singh 2016** [21]	Observational retrospective	Monocenter	40	56.8 ± 13.9	Anal	APR	Gracilis	23.6
**Burke 1995** [38]	Observational retrospective	Monocenter	18	55 (33–79)	Vaginal, anal	PE	Gracilis	25 (2–60)

Footnote: APR, Abdominoperineal resection; PE, Pelvic exenteration; NS, not specified.

### 3.2. Overall Wound Complications

**Figure 2:**
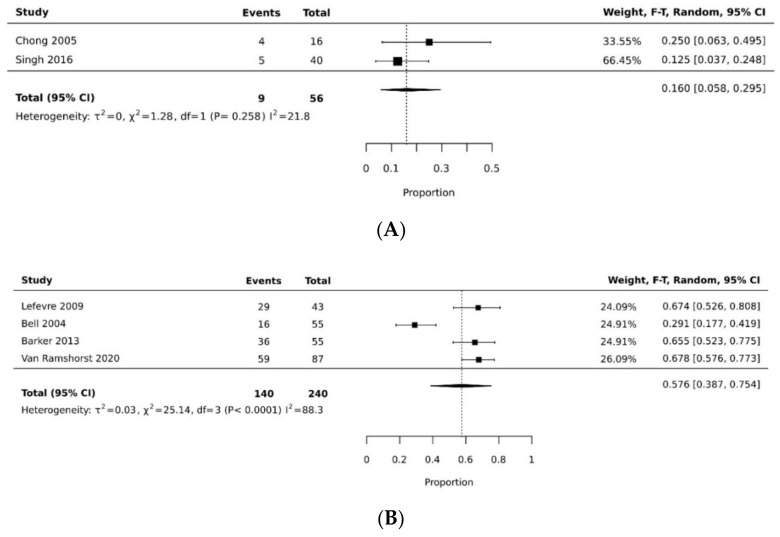
(**A**) Forest plot of gracilis overall donor site complication rate [21,35]. (**B**) Forest plot of VRAM overall donor site complication rate [11,27,29,32].

**Figure 3:**
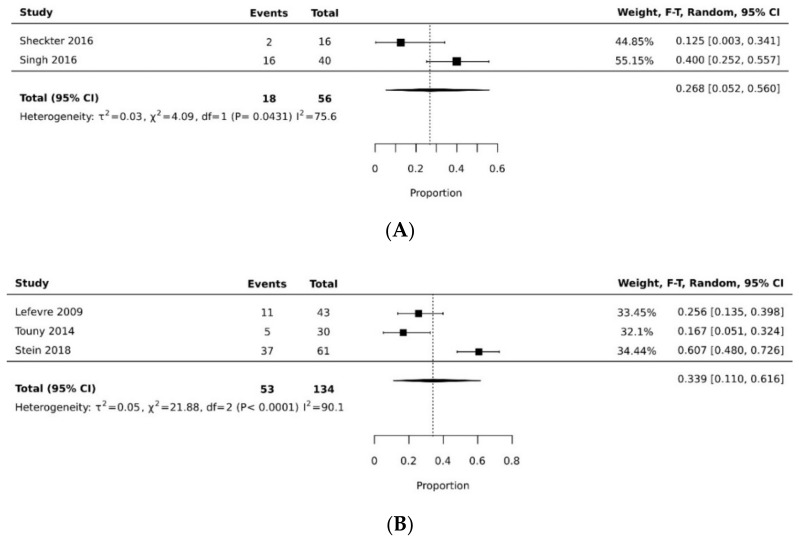
(**A**) Forest plot of gracilis overall recipient site complication rate [21,37]. (**B**) Forest plot of VRAM overall recipient site complication rate [26,31,32].

In the proportional meta-analysis, the pooled resolution rate of overall donor site complications for VRAM flap (0.576 [95% CI 0.387, 0.754]) was significantly higher than the pooled resolution rate of overall donor site complications of gracilis flap (pooled proportion = 0.160 [95% CI 0.058, 0.295]), with a statistically significant difference (*p* < 0.05). [11,21,27,29,32,35] (Figure 2A,B, Table 3). The pooled resolution rate of overall recipient site complications for VRAM (0.339 [95% CI 0.110, 0.616]) did not differ significantly from gracilis (0.268 [95% CI 0.052, 0.560]) [21,26,31,32,37] (Figure 3A,B).

**Table 3:** Comparison of clinical outcomes between VRAM and gracilis.

	VRAM		Gracilis		Z-Test	
Outcome	Proportion	n	Proportion	N	z	*p*-Value
**Total flap failure**	0.025	628	0.064	43	−1.513	0.131
**Partial flap failure**	0.061	494	0.087	110	−0.995	0.317
**Recipient site dehiscence**	0.158	713	0.276	85	−2.734	<0.05
**Donor site dehiscence**	0.076	435	0.160	56	−2.115	<0.05
**Donor site infection**	0.091	642	0.078	110	0.442	0.660
**Recipient site infection**	0.104	575	0.135	108	−0.949	0.342
**Overall recipient site complications**	0.339	134	0.268	56	0.958	0.337
**Overall donor site complications**	0.576	240	0.160	56	5.606	<0.05

### 3.3. Surgical Outcomes

**Figure 4:**
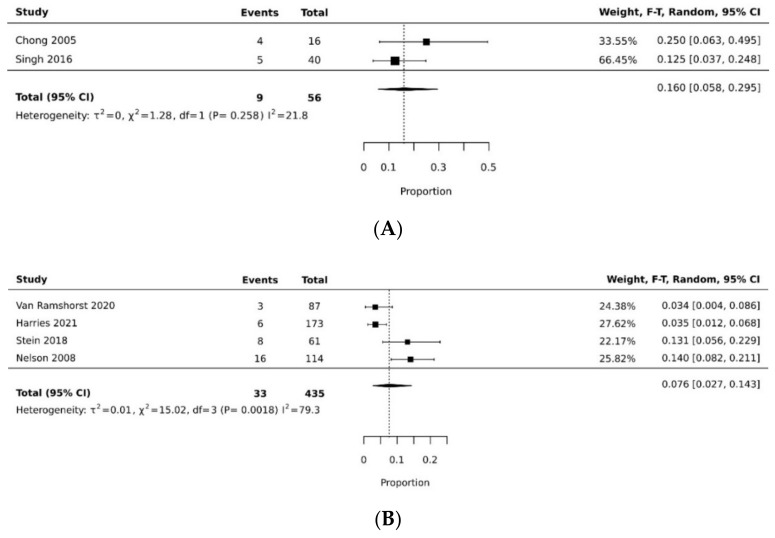
(**A**) Forest plot for donor site dehiscence for gracilis [21,35]. (**B**) Forest plot for donor site dehiscence for VRAM [26,29,30,34].

**Figure 5:**
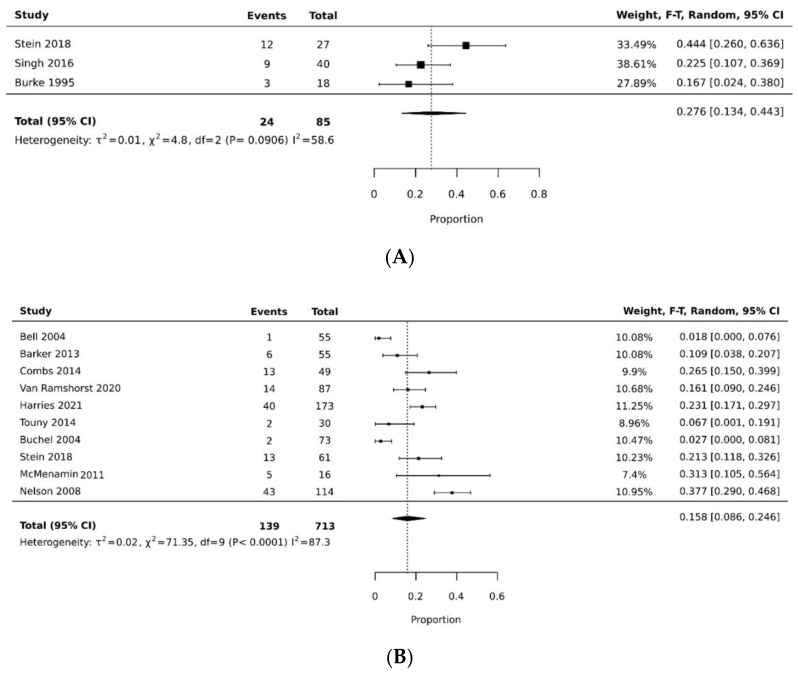
(**A**) Forest plot for recipient site dehiscence for gracilis [21,26,38]. (**B**) Forest plot for recipient site dehiscence for VRAM [11,12,26,27,28,29,30,31,33,34].

**Figure 6:**
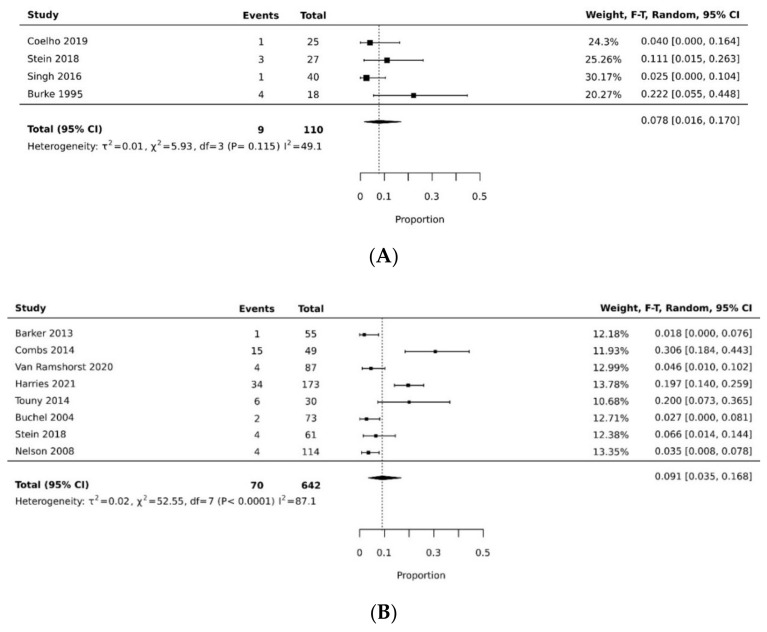
(**A**) Forest plot for donor site infection rate for gracilis [21,26,36,38]. (**B**) Forest plot for donor site infection rate for VRAM [12,26,27,28,29,30,31,34].

**Figure 7:**
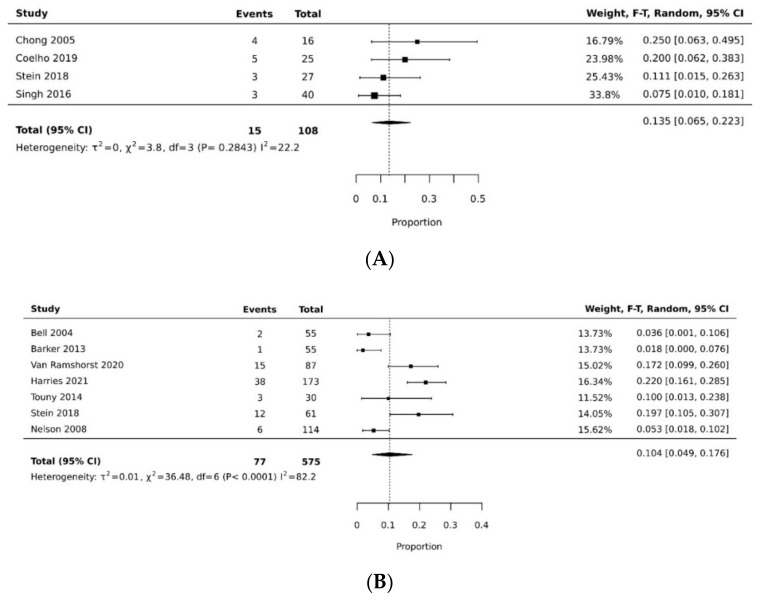
(**A**) Forest plot for recipient site infection rate for gracilis [21,26,35,36]. (**B**) Forest plot for recipient site infection rate for VRAM [11,26,27,29,30,31,34].

**Figure 8:**
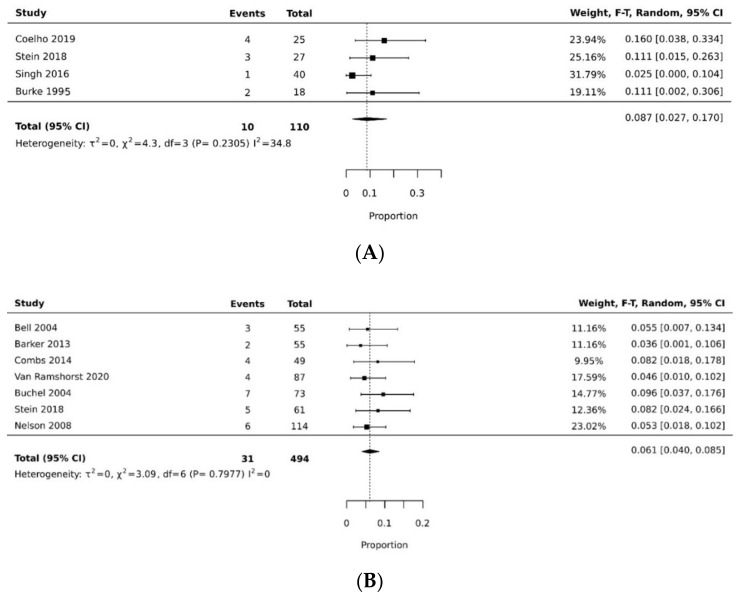
(**A**) Forest plot for partial flap necrosis for gracilis [21,26,36,38]. (**B**) Forest plot for partial flap necrosis for VRAM [11,12,26,27,28,29,34].

**Figure 9:**
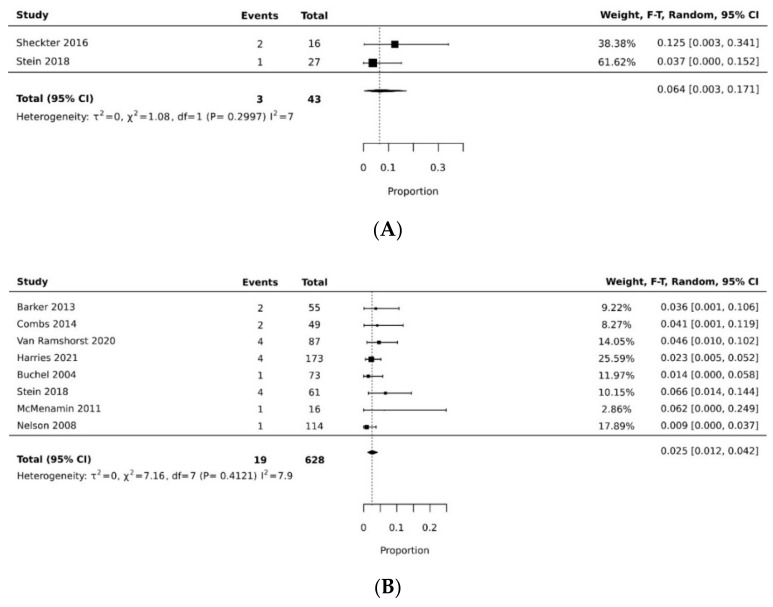
(**A**) Forest plot for total flap necrosis for gracilis [26,37]. (**B**) Forest plot for total flap necrosis for VRAM [12,26,27,28,29,30,33,34].

**Figure 10:**
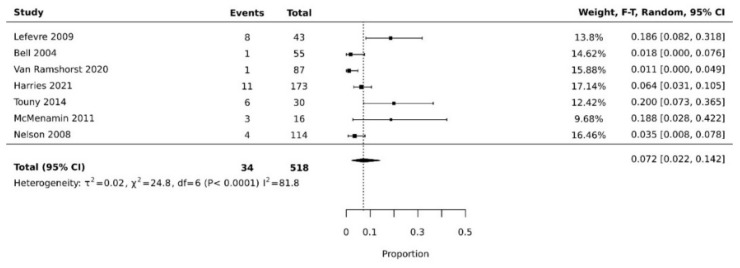
Forest plot for donor site herniation for VRAM [11,29,30,31,32,33,34].

The pooled rate of donor site dehiscence for gracilis flap (0.160 [95% CI 0.058, 0.295]) was higher than that for the VRAM flap (0.076 [95% CI 0.027, 0.143]) (*p* < 0.05) [21,26,29,30,34,35] (Figure 4A,B). The pooled rate of recipient site dehiscence with the gracilis (0.276 [95% CI 0.134, 0.443]) was higher than that of the VRAM (0.158 [95% CI 0.086, 0.246]) [11,12,21,26,27,28,29,30,31,33,34,38] (Figure 5A,B). Concerning donor site infection rate, the pooled analysis was similar for both flaps: VRAM (0.091 [95% CI 0.035, 0.168]) vs. gracilis (0.078 [95% CI 0.016, 0.170]) [12,21,26,27,28,29,30,31,34,36,38] (Figure 6A,B). Recipient site infection rate was similar between gracilis (0.135 [95% CI 0.065, 0.223]) and VRAM (0.104 [95% CI 0.049, 0.176]) [11,21,26,27,29,30,31,34,35,36] (Figure 7A,B). Partial flap necrosis was similar with gracilis (0.087 [95% CI 0.027, 0.170]) and VRAM (0.061 [95% CI 0.040, 0.085]) [11,12,21,26,27,28,29,34,36,38] (Figure 8A,B). No statistical difference in terms of total flap necrosis was seen between gracilis reconstruction (0.064 [95% CI 0.003, 0.171]) and VRAM (0.025 [95% CI 0.012, 0.042]) [12,26,27,28,29,30,33,34,37] (Figure 9A,B). VRAM donor site herniation was estimated at 0.072 [95% CI 0.022–0.142] [11,29,30,31,32,33,34] (Figure 10).

## 4. Discussion

This meta-analysis is to our knowledge the first pooled analysis of surgical outcomes between VRAM and gracilis pelvic reconstruction following oncologic resection.

Multiple surgical options for closure of perineal defects have been described: primary closure, use of acellular dermal matrix, loco-regional flaps, and pedicled muscular flaps. Two studies demonstrated the superiority of muscular flaps compared to primary closure [1,17]. VRAM and gracilis are two popular muscle flaps used in perineal reconstruction, motivating their comparison in this meta-analysis. Our findings suggest that both flaps can safely be used for vulvo-perineal reconstruction. They both provide healthy, well-vascularized tissues that can fill the resection dead space and minimize complication rates.

Our analysis showed the pooled resolution rate of overall donor site complications for VRAM flaps was significantly higher than gracilis flaps. This might be due to multiple factors, among which is the robust nature of the flap [39,40]. Classically the VRAM flap consists of a 5 to 10 cm-wide skin paddle designed vertically above the right rectus abdominis muscle. Normally, the anterior rectus sheath fascia is incised, and the rectus muscle and overlying soft tissue are elevated away from the posterior rectus sheath [41]. This can weaken the abdominal wall, leading to more complications. Donor site herniation is specific to VRAM, explaining the potentially higher overall donor site complications compared to gracilis (Figure 10). Although most of the authors report an important number of events, Bell et al. had a lower proportion of donor site complication than the three other authors. In their series, they modified the technique described by Taylor et al., who in 1983 described the vertical rectus abdominis myocutaneous flap consisting of a large paddle of skin and the underlying rectus abdominis muscle vascularized by the deep inferior epigastric vessels and passed through the pelvis to the perineum [20]. They modified the skin paddle to include an oblique skin paddle rather than the vertical skin paddle, defending the oblique skin paddle as having the following advantage: the laxity of the abdominal skin is more significant in the oblique direction than in the vertical direction, thus allowing a larger paddle and minimizing tension at the donor site. This could explain the decreased donor site morbidity in their series as compared to the other authors, who instead used a vertical skin paddle.

Most of the authors did not report the size of their skin paddle in the VRAM flap, not allowing an analysis of the correlation between skin paddle size and complication rate.

The pooled rate of overall recipient site complications for VRAM was relatively similar to that of gracilis, with no statistically significant difference. However, this outcome was reported only in a few studies. Recipient site complications for gracilis flap seems to be dependent on the inclusion of a skin island (myocutaneous). In their study, Nelson et al. reported an increased complication rate with gracilis myocutaneous flap when compared with VRAM flap for reconstruction of pelvic defects, while Chong et al. demonstrated successful reconstruction of pelvic defects using gracilis muscle flaps and re-approximation of the perineal skin [25,39]. Most authors in our study used the gracilis as a muscular flap without a skin paddle for reconstruction with perineal skin approximation, explaining the similar overall recipient site complication with the VRAM flap [40,42].

When more specifically assessing recipient site dehiscence, it was higher for gracilis than VRAM flap (*p* < 0.05). The robust and voluminous nature of the VRAM flap is an added value compared to the gracilis, which may be more exposed to tension, hence favoring dehiscence at the recipient site. The dehiscence rate might be worsened if the gracilis flap is harvested without a skin paddle. Inside the VRAM group, we noticed that Bell et al. had the lowest recipient site dehiscence rate. This can be explained with the use of the ORAM design, which provides a large skin paddle, allowing tension-free closure of the resection site [11]. However, we note some variability between other studies using the classical VRAM design, with Buchel et al. and Tunny et al. recording lower recipient site dehiscence than the other studies [12,31]. This might potentially be explained by a larger harvested skin paddle. However, as skin paddle size was not reported in those studies, it is not possible to affirm this possibility.

In our meta-analysis, no significant difference emerged concerning the donor site and recipient site infection rate between both flap types. This might be explained because both flaps are of a muscular nature, therefore providing a good vascular supply to the injured area [33].

While our results suggest similar outcomes between both flap types, this meta-analysis might be impacted by several limitations. It included only one RCT and not enough comparative studies. Ideally, a meta-analysis of RCTs or comparative studies would provide stronger evidence than a proportional meta-analysis because the primary aim of the selected studies is to describe the evolution of a cohort and not to compare it between two interventions, meaning populations and interventions might differ significantly, inducing potential bias in the analysis.

Furthermore, outcome definitions reported in the studies are not sufficiently described, meaning complication rates might be under/overestimated. To limit those potential biases, we decided to use the outcomes as reported in the studies.

Furthermore, heterogeneity was high for most of the outcomes, depicting the diversity of the studied populations and interventions. We demonstrate that partial flap necrosis and total flap necrosis was similar for both flaps, as those are major reconstructive outcomes. One major criticism of the gracilis flap from certain authors is the questionable viability of the cutaneous paddle when used as a myocutaneous flap due to inconsistent perforator supply. Some prefer to use a muscle-only gracilis flap and obtain tension-free skin closure with a V-Y or bilobed flap if necessary [21].

Muscular flaps are considered a safe reconstructive option following oncologic resection for vulvo-perineal defects. Devulapalli et al. compared primary closure vs. flap closure in their meta-analysis, where they strongly validate the use of myocutaneous flaps for reducing perineal morbidity, particularly in patients with prior irradiation to the pelvis [1]. We demonstrated that VRAM and gracilis flaps, which are popular muscular flaps, are both safe according to our findings. Results should be interpreted cautiously due to potential bias linked to study design and high heterogeneity between study populations. Flap choice should be made according to a patient’s characteristics and needs, but also according to surgeon experience.

## 5. Conclusions

We demonstrated that both flaps can be used safely for vulvo-perineal reconstruction following oncologic resection with similar recipient site outcomes, although the VRAM flap has more donor site complications than the gracilis flap.

## Figures and Tables

**Table 1:** Inclusion/exclusion criteria.

PICOS	Inclusion	Exclusion
**Population**	Adults with vulvo-perineal reconstruction with VRAM or gracilis flap	Cadaveric, animal studies
**Intervention**	Pedicled VRAM or gracilis flap for vulvo-perineal reconstruction following oncologic resection	Other flaps for vulvo-perineal reconstruction
**Comparator**	The study analysis compared postoperative clinical outcome parameters	
**Outcomes**	Main outcomes: infection rate, dehiscence, partial or total flap necrosis, length of hospital stay (in days) and follow up period (months)	Studies that don’t report main outcome
**Study design**	Comparative studies Case series	Reviews, meta-analysis, case reports, Unpublished studies
